# Cytoplasmic-genetic male sterility gene provides direct evidence for some hybrid rice recently evolving into weedy rice

**DOI:** 10.1038/srep10591

**Published:** 2015-05-27

**Authors:** Jingxu Zhang, Zuomei Lu, Weimin Dai, Xiaoling Song, Yufa Peng, Bernal E. Valverde, Sheng Qiang

**Affiliations:** 1 Weed Research Laboratory of Nanjing Agricultural University, No.1 Weigang, Xuanwu District, Nanjing 210095, China; 2 State Key Laboratory of Crop Genetics &Germplasm Enhancement, Nanjing Agricultural University, No.1 Weigang, Xuanwu District, Nanjing 210095, China; 3 Institute of Plant Protection, China Academy of Agricultural Sciences, No.2 Yuanmingyuan West Road, Beijing 100193,China; 4 Faculty of Life Sciences, The University of Copenhagen, Hojebakkegaard Allé 13, Taastrup, DK-2630, Denmark

## Abstract

Weedy rice infests paddy fields worldwide at an alarmingly increasing rate. There is substantial evidence indicating that many weedy rice forms originated from or are closely related to cultivated rice. There is suspicion that the outbreak of weedy rice in China may be related to widely grown hybrid rice due to its heterosis and the diversity of its progeny, but this notion remains unsupported by direct evidence. We screened weedy rice accessions by both genetic and molecular marker tests for the cytoplasmic male sterility (CMS) genes (Wild abortive, WA, and Boro type, BT) most widely used in the production of *indica* and *japonica* three-line hybrid rice as a diagnostic trait of direct parenthood. Sixteen weedy rice accessions of the 358 tested (4.5%) contained the CMS-WA gene; none contained the CMS-BT gene. These 16 accessions represent weedy rices recently evolved from maternal hybrid rice derivatives, given the primarily maternal inheritance of this trait. Our results provide key direct evidence that hybrid rice can be involved in the evolution of some weedy rice accessions, but is not a primary factor in the recent outbreak of weedy rice in China.

Weedy rice (*Oryza sativa*) has strong seed-shattering and seed dormancy and is highly competitive with cultivated rice (*O. sativa*). At present, there is no effective and selective chemical control available to cull the weedy rice population, given that both cultivated and weedy rice are the same species and share similar morphological and physiological characteristics^1^,^2^. Weedy rice has therefore become one of the most harmful weeds in paddy fields^3^. Based on the analysis of biological characteristics, molecular markers, and specific genes in plants from different areas worldwide, it has been proposed that weedy rice originated as hybrid progeny of wild rice (*Oryza rufipogon*) and cultivated rice^4^,^5^,^6^, de-domestication or reversion of cultivated rice towards the wild type^7^,^8^, or as hybrid progeny of *indica-japonica* subspecies^9^. The origin and evolution pattern of weedy rice most likely differs among plants growing in different areas and should be explored. Weedy rice has become a serious problem in China. Comparative studies of the morphological characteristics and genetic diversity of Chinese weedy rice indicate that it is closely related to companion cultivated rice and may indeed have originated by the reversion of cultivated rice or by gene introgression among rice varieties^10^,^11^,^12^,^13^. We here provide new compelling evidence of the involvement of cultivated rice, in our case, hybrid rice, directly in the evolution of some weedy rice accessions.

Gene flow and introgression are driving forces for natural and cultivated plant evolution. They frequently and widely occur among cultivars, wild relatives, and compatible weedy types. These natural phenomena have existed for thousands of years and contribute to the evolutionary history of crops and their wild relatives^14^. Many studies have reported that gene flow can occur among wild rice, cultivated rice and hybrid rice accessions^10^,^15^,^16^. Existing evidence depicts strong relationships between cultivars and weedy rices at local levels^10^,^12^,^13^,^17^ to which our study contributes further proof that cultivated hybrid rice may directly participate in the evolution of weedy rice.

Rice has been cultivated in China for thousands of years but the spread of weedy rice is relatively recent. Coincidentally, hybrid rice rapidly became popular in China in the 1970 s, and by the 1990 s accounted for approximately half of the area of cultivated rice. Currently, some 17 million ha of hybrid rice are grown in China, representing 80% and 3% of its cultivated *indica* and *japonica* rice, respectively^18^,^19^,^20^,^21^. Unlike the other two forms of rice widely cultivated in China - landraces (local varieties selected and maintained by farmers) and conventional varieties (those improved by breeders) - the continued production of which depends on their autogamy, hybrid rice is instead the heterotic F_1_ generation of crosses between genetically distant parents. Thus, characteristics of hybrid rice progeny often strongly segregate owing to the great genetic diversity of parental lines^18^. Hybrid rice progeny that escapes harvesting may segregate into distinct biotypes through gene recombination, some of which might evolve into weedy rice. Interestingly, Jiangsu province is currently experiencing a serious infestation of weedy rice, most of which is of the *indica*-type despite the fact that *japonica* conventional varieties have been the primary type of cultivated rice in this region for more than 20 years^12^,^13^. However, *indica*-type hybrid rice was planted on a large scale in this province in past decades. It is suspected that weedy rice in some areas of Jiangsu province may have evolved from segregating progeny of *indica* hybrid rice, which is supported by results from genetic and morphological analyses^12^,^22^. Despite the widespread planting of hybrid rice, little is known about the contributions of its segregating volunteers to the ongoing evolution of weedy rice. Resolving the evolutionary origin of weedy rice in areas of hybrid rice cropping is particularly relevant, as any involvement of hybrid rice in the spread of weedy rice would pose challenges to the sustainability and efficiency of hybrid rice technology.

Hybrid rice is obtained by either three-line or two-line systems. The former was developed earlier and became widely grown, achieving more than 90% of the hectarage dedicated to hybrid rice between 1976 and 2000. Since then, its proportion has decreased only slightly to approximately 80%^23^. The female parent of three-line hybrid rice is a cytoplasmic male sterile line, whereas the male parent is a restorer line. The hybrid contains cytoplasmic male sterility (CMS) genes and the heterozygous restorer of fertility (Rf) genes. The male-sterile female parent can be traced by cytoplasmic genotype analysis due to maternal inheritance of the sterility gene^24^. Although the potential for a minor paternal transmission of the mitochondrial genome has been recognized in several species^25^,^26^ and the ultrastructure of the rice sperm cell shows mitochondria localized mainly in the head of the sperm cell^27^, organelle DNA was not detected^28^,^29^. Thus, it is reasonable to assume that the CMS gene is primarily maternally inherited. If weedy rice directly evolved as progeny of hybrid rice, weedy rice individuals should contain a CMS gene regardless of any involvement of gene flow during the evolution process. This offers a unique way to verify the direct involvement of hybrid rice in the origin of some weedy rice types.

A wild abortive (WA) cytoplasmic male sterile line was first developed and became the most widely used to breed *indica* hybrid rice. A Boro type (BT) cytoplasmic male sterile line is the most widely used to breed *japonica* hybrid rice^20^. To test the hypothesis that some weedy rice accessions may have directly originated from hybrid rice, we used the CMS-WA and CMS-BT genes as markers to test the lineage of a collection of weedy rice samples. These genes are largely maternally inherited and are therefore not expected to be transferred to weedy rice through pollen-mediated gene flow.

## Results

### *Indica*-*japonica* subspecies classification and cross-compatibility of weedy rice accessions

**Figure 1:**
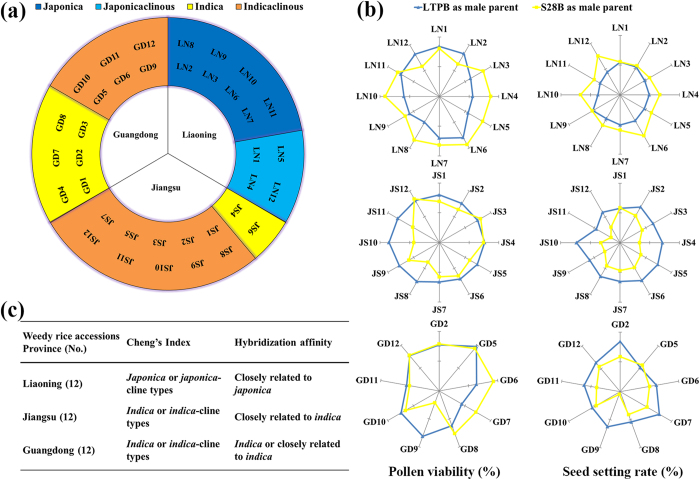
Subspecies classification of 36 weedy rice accessions and their cross compatibility with typical *indica* and *japonica* rice. (**a**) Classification of 36 weedy rice accessions collected in three Chinese provinces according to their Cheng’s Index^30^. (**b**) Hybrid progeny fertility comparison of 36 weedy rice accessions (♀) and two maintainer lines (♂). LTPB as male parent (typical *indica*), S28B as male parent (typical *japonica*). Percent pollen viability (left radar charts) and percent seed setting rate (right radar charts) of hybrid progeny between weedy rices from Liaoning (LN), Jiangsu (JS) and Guangdong (GD) provinces as female parents and S28B or LTPB maintainer lines as male parents. (**c**) Summary of subspecies classification of 36 weedy rice accessions by Cheng’s Index and hybridization affinity method.

Prior to assaying 322 weedy rice accessions collected across China for CMS genes, 36 accessions independently obtained from three major rice-producing provinces experiencing serious weedy rice infestations (Liaoning, Jiangsu and Guangdong) were assigned to *indica* or *japonica* subspecies according to Cheng’s Index, which measures six rice characteristics^30^. This allowed us to rule out infertility associated with *indica*-*japonica* hybridization. Of the 12 weedy rice samples from Liaoning province, eight accessions were *japonica* and four were *japonica*-cline types. Conversely, of the 24 weedy rice accessions from Jiangsu and Guangdong, eight accessions were *indica* and 16 were *indica*-cline type (Fig. 1a). Thus, there was regional differentiation in *indica*-*japonica* subspecies. Two-thirds of the weedy rice samples (i.e., those identified as *japonica*- and *indica*-cline types) can be considered intermediate forms between *indica* and *japonica* rices.

Sixty-nine hybrid progeny were obtained from 72 artificial crosses between the 36 weedy rice (WR) accessions and either the *indica*-type WA and *japonica*-type BT maintainer lines LTPB and S28B, respectively, as pollen donors, (36 WR × LTPB, S28B). No hybrids were obtained from the combinations of three accessions from Guangdong (GD) with the S28B line (GD1,GD3,GD4 × S28B) due to seed abortion before grain filling. All the obtained hybrid progeny were fertile according to affinity detection tests except for GD9 × S28B, which had very low fertility (Fig. 1b). Therefore, most combinations (all weedy rice × LTPB and most weedy rice × S28B) were cross-compatible, with the exception of the combinations of four of the GD weedy rices × S28B. The cross-incompatibility observed in these four combinations indicates that GD1, GD3, GD4 and GD9 belong to the *indica* subspecies. Weedy rice from Liaoning (LN) hybridized better with S28B than with LTPB, reinforcing the notion that weedy rice from this region is closely related to *japonica*. Conversely, weedy rice accessions from Jiangsu (JS) and Guangdong were apparently more closely related to *indica* because hybridization was more effective with LTPB than with S28B. Most weedy rice accessions, however, were widely compatible because they were cross-compatible with both typical *japonica* and *indica* rice, and the difference between the affinities was not considerable. The classification of weedy accessions based on hybridization affinity was consistent with the classification obtained with Cheng’s indices (Fig. 1c).

### The relationship between weedy rice and BT hybrid rice

The seed setting rates of the hybrid progeny of weedy rice accessions and the sterile line S28A as the female parent (S28A × 36WR) were all less than 5%, which suggested that these weedy rice accessions lack the Rf gene for CMS-BT. It is unlikely that they also have the CMS-BT because they set seed normally. As artificial (GD1, GD2, GD3, GD9 × S28B) hybridizations were cross-incompatible, and given that maintainer line S28B shares the same nuclear constitution as S28A, the infertility of these hybrid progeny might involve other factors in addition to the absence of the Rf genes for CMS-BT in the weedy rice parents.

Assay of pollen viability in hybrid progeny (S28A × 36WR) by I_2_-KI solution was not sufficiently clear to ensure accuracy, because the pollen of BT type male sterile lines typically has spherical abortion characteristics or a stained abortion phenotype. The size and staining depth of pollen grains were diversiform (Supplementary Figure S1).

**Figure 2:**
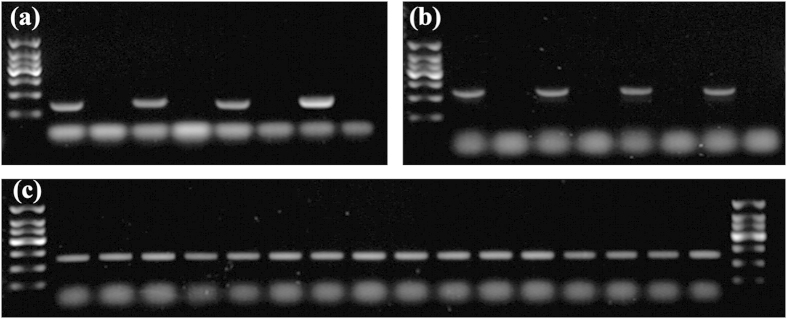
Identification of cytoplasmic male sterility (CMS) genes in sterile lines, maintainer lines and weedy rice accessions. (**a**) Marker p3p4 can only amplify a 239 bp fragment from the Boro type (BT) sterile line but not from the maintainer line. From left to right are Marker (150 bp DNA ladder), S28A, S28B, LiuqianxinA, LiuqianxinB, 863A, 863B, XuIIA, and XuIIB. (**b**) Marker *cms* can only amplify a 386 bp fragment from the wild abortive (WA) type sterile line but not from the maintainer line. From left to right are Marker (150 bp DNA ladder), Zhenshan97A, Zhenshan97B, Tianfeng A, Tianfeng B, Zhenpin A, Zhenpin B, LTPA, and LTPB. (**c**) Sixteen weedy rice accessions that were confirmed to contain CMS-WA. From left to right are Marker (150 bp DNA ladder), WRJS026-01 (Jiangsu), WRHA016-01, WRHA016-03 (Hainan), GD9, GD10, GD11, GD12, WRGD012-04, WRGD013-06, WRGD013-13, WRGD013-17, WRGD022-03, WRGD022-06, WRGD022-09, WRGD022-10, WRGD022-13 (Guangdong), and Marker (150 bp DNA ladder).

The reliability of the p3p4 marker, specific for CMS-BT, was verified and it was determined that it only amplified with the DNA fragment from material containing the CMS-BT gene (Fig. 2a). None of the 36 weedy rice accessions contained the CMS-BT gene, according to assay of the p3p4 marker. These results are consistent and suggest that these weedy rice accessions are not genetically related to BT hybrid rice.

### Relationship between weedy rice and WA type hybrid rice

**Figure 3:**
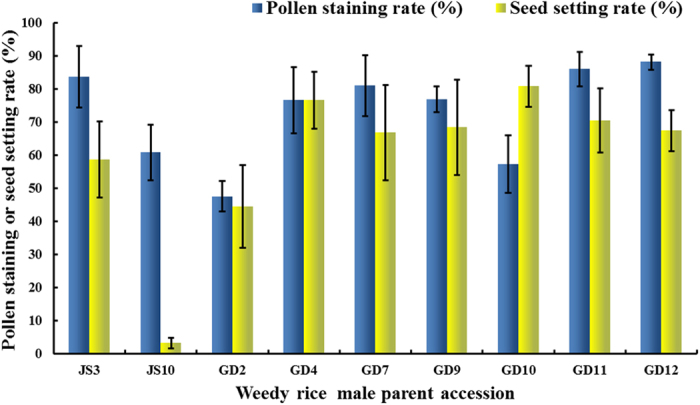
Assessment of fertility of progeny of synthetic hybrids between LTPA (♀) and weedy rice accessions (). The error bars denote ± SD.

Nine hybrid combinations between the male-sterile line LTPA as female parent and weedy rices JS3 and JS10 (Jiangsu) and GD2, GD4, GD7, GD9, GD10, GD11, and GD12 (Guangdong) as male parents were fertile as determined by pollen viability and seed setting of their hybrid progenies (LTPA × 36WR; Fig. 3). The infertility of the remaining hybrid progenies must have been under the control of a CMS-WA gene (weedy rice does not contain the Rf genes for CMS-WA). No false negatives existed; this was concluded because LPTB shares the same nuclear constitution with LTPA, and there was no cross-incompatibility in the artificial 36WRxLTPB hybridizations. Therefore, the nine weedy rice parents must have contained the Rf gene for CMS-WA (abbreviated to R-WA) to produce fertile hybrids with LTPA. Thus, they were subjected to further analyses to determine whether they also carried the CMS-WA.

**Figure 4:**
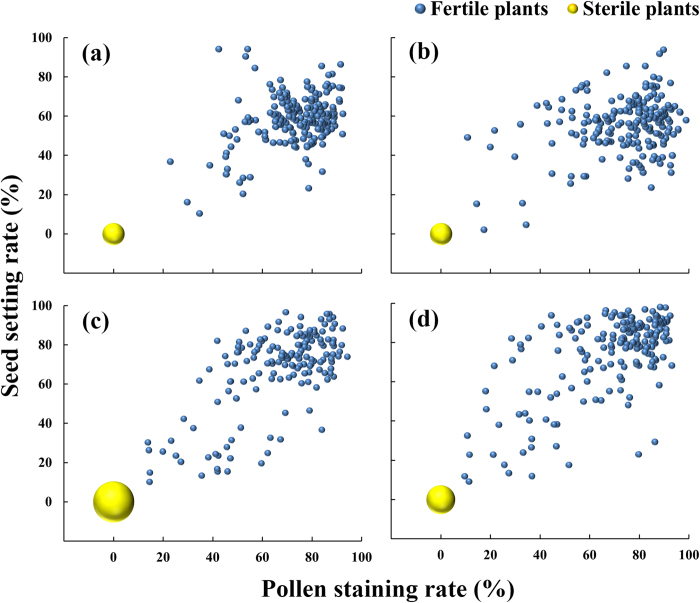
Assessment of fertility segregation in F_2_ progeny of hybrids whose male parent was the maintainer line LTPB and with the weedy rice accessions GD9 (**a**), GD10 (**b**), GD11 (**c**), or GD12 (**d**) as female parent. Each blue dot represents a fertile plant; the total number of fertile plants were 196 (**a**), 202 (**b**), 160 (**c**), 185 (**d**). The yellow dots represent sterile plants, with different sizes corresponding to the number of sterile plants: 11 (**a**), 11 (**b**), 41 (**c**) and 19 (**d**).

Four combinations of the nine F_2_ hybrids (9 R-WA × LTPB) of interest, segregated sterile plants whose female parents were GD9, GD10, GD11, GD12 (Fig. 4). The F_2_ hybrid progeny with GD11 as the female parent conformed to a 1:3 ratio of sterile plants to fertile plants according to a chi-square test for segregation ratio, and those for which the female parents were GD9, GD10, and GD12 exhibited a 1:15 ratio. Different segregation ratios could be attributed to the weedy rice accessions containing one or two main effect Rf genes for CMS-WA. This result confirmed that weedy rice accessions GD9, GD10, GD11, and GD12 contained CMS-WA and had a strict direct relation to WA type hybrid rice. Coincidentally, the cultivated rice accessions grown together with these four weedy rice lines were three-line hybrids containing CMS-WA.

### Identification of wild abortive cytoplasmic male sterility in other weedy rice accessions

**Figure 5:**
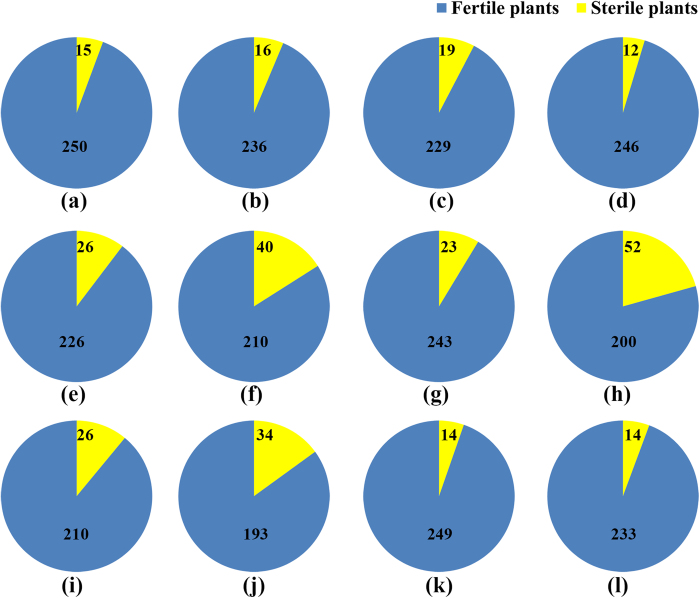
Fertility segregation proportions observed in F_2_ progeny populations. The numbers represent the number of fertile or sterile plants. (a-l) represent the F_2_ hybrid progenies of LTPB as male parent, and (in letter order) the following weedy rices as female parents: WRJS026-01 (Jiangsu), WRHA016-01 and WRHA016-03 (Hainan), WRGD012-04, WRGD013-06, WRGD013-13, WRGD013-17, WRGD022-03, WRGD022-06, WRGD022-09, WRGD022-10, and WRGD022-13 (Guangdong). Designations of the weedy rice accessions are those of the weedy rice germplasm bank of NJAU.

Putative *cms* marker (specific marker to detect CMS-WA) was considered validated if it reliably yielded a band only for the CMS-WA gene that was contained by the plant (Fig. 2b). According to an assay for the *cms* marker, 12 of the 322 additional weedy rice accessions contained CMS-WA (Fig. 2c). One of these 12 accessions was from Jiangsu province, two accessions were from one population from Hainan province, and nine accessions were from three populations from Guangdong province. These results were further verified by artificial hybridization as described above. All progeny of the 12 F_2_ hybrids tested (12WR containing CMS-WA detected with the *cms* marker × LTPB) segregated sterile plants (Fig. 5). These results are consistent and confirm that these accessions contained CMS-WA and were genetically related to a WA-type hybrid rice.

### Corroboration of results

During the process of preparing this manuscript, the gene *WA352* that is responsible for CMS-WA was cloned^31^. Using specific marker orWA352 based on *WA352* to detect the possible presence of CMS-WA in all 358 weedy rice samples, we unequivocally corroborated our previous findings with the *cms* marker: the same 16 of the 358 weedy rice samples were confirmed as having CMS-WA (Supplementary Figure S2). Furthermore, we sequenced the 16 amplified products and consistently found them containing the *WA352* gene.

## Discussion

### Subspecies affiliation of selected weedy rice accessions and their relationship to hybrid rice

Individuals from a selected collection of 36 weedy rice accessions - 12 from each of three Chinese provinces - were assigned to *indica* and *japonica* subspecies and used to validate experimental methods. All weedy rice accessions from Liaoning, where *japonica* rice is predominantly grown, belonged to this subspecies (eight accessions) or were identified as *japonica*-cline (four accessions) but none were derived from hybrid rice. Previous studies have determined that weedy rice accessions from this northern province are genetically narrowly diverse (within populations) and are closely related to cultivated *japonica* rice but distant to *indica* rice^11^,^13^,^17^. They probably originate from local varieties through de-domestication and hybridization^13^,^17^. Our results are consistent with previous studies despite the limited sample size, and they rule out the involvement of hybrid rice in the origin of this province’s weedy rice. The weedy rice accessions from the other two provinces, Jiangsu and Guandong, were all *indica* (although predominantly *indica*-cline), also in agreement with previous studies^12^,^13^. Currently, rice production in Jiangsu is primarily of *japonica* cultivars^12^, although *indica* rice has been grown in the past and is still planted adjacent to *japonica* fields by some farmers. These conditions may help explain the intermediate nature of a portion of the tested weedy rices, which may have evolved through gene flow and hybridization. In Guangdong, where weedy rice is a serious problem exacerbated by increasing adoption of direct seeding^32^, cultivated rice is primarily of the *indica* type. Weedy rice most likely originated from local cultivated rice and not from the *japonica*-type wild rice found in the province^13^. Moreover, four weedy rice accessions from Guangdong - GD9, GD10, GD11, and GD12 - contained CMS-WA and were considered direct descendants of WA type hybrid rice.

### Hybrid rice has participated in the evolution of some weedy rice in China

Sixteen weedy rices (one from Jiangsu, two from Hainan, and nine from Guangdong), representing 4.5% of the total 358 accessions analyzed, contained CMS-WA. The accuracy of this finding is supported by both genetic and molecular marker tests. The CMS-WA that was originally selected from a sterile mutant wild rice plant (*O. rufipogon*) has been exploited in the production of the majority of three-line *indica*-type hybrid rice cultivars in China^20^. Hybrid rice containing CMS-WA began occupying increasing proportions of the area dedicated to cultivating rice in the 1970 s. The sterile phenotype in wild rice is extremely rare because the CMS gene, although probably present in a relative high proportion (up to 5%) of the wild individuals^31^, is overcome by the restoration of fertility by Rf; the finding of the original sterile individual was indeed a major breakthrough in rice breeding. For wild rice to be a source of the CMS-WA gene the individuals carrying it should have participated as female parents in crosses with cultivated forms to generate weedy types. A recently study has found a lack of relationship of weedy rice with wild rice in areas where *O. rufipogon* is still present^13^. This suggests that the CMS-WA in weedy rice most likely was acquired recently (within the last 40 years) from hybrid rice. Because this gene is coded by the mitochondrial genome^31^, it is primarily maternally inherited^24^,^28^,^29^, indicating that the original hybrid parent was likely female. This provided direct evidence that hybrid rice has been involved in the evolution of some weedy rice accessions.

The majority of the 358 accessions came from the three provinces (Guangdong, Hainan, and Jiangsu) where weedy rice infestations are most severe. However, given the considerable number of accessions tested and their disparate geographical origins, it appears that weedy rices directly related to hybrid rice are a relatively small proportion of those currently infesting rice fields in China. There is a chance that the CMS trait is unfit for weedy rice and segregates out of weedy populations, further reducing the frequency of weedy rice individuals carrying the CMS gene. However, the CMS-WA phenotype can be restored by either of two non-allelic, dominant Rf genes^31^. Thus for hybrid rice individuals containing one or two heterozygous Rf genes, the expected proportion of sterile individuals among its volunteers (the F_2_ generation) would theoretically fall between 0.25 (3:1) and 0.062 (15:1). If the two Rf genes become homozygous or if there is a sufficient load of pollen containing Rf genes from cultivated or weedy rice (frequent in regions of hybrid rice cultivation), plants carrying the CMS-WA will become fertile and consequently the unfitness conferred by CMS-WA would be relieved. This would rule out the notion that the outbreak of weedy rice in China can be attributed to the ample cultivation of hybrid rice. Although CMS-BT has been exploited in *japonica* hybrid rice production, it was not detected in any of the 36 weedy rice accessions from Liaoning, Jiangsu and Guangdong. The area dedicated to cultivating BT-type hybrid rice is limited, thus decreasing the probability of detecting weedy rice accessions containing the CMS-BT.

### Possible evolutionary processes leading to weedy rice

The evolution of some weedy rice accessions from cultivated hybrid rice has probably been a complex process. The CMS-WA in weedy rice could have come from hybrid rice through direct or indirect evolution.

Direct evolution entails the possibility that some individuals of segregant populations of hybrid rice becoming weedy rice through gene recombination. The heterosis in hybrid rice can be considered only transitory in the scheme of weedy rice evolution, but gene recombination in progeny should have a long-standing effect on adaptation to novel environments by the creation of new gene combinations upon which selection can act. A case study has shown that hybrid progeny of different rice varieties could evolve into weedy rice like individuals, and their occurrence was more frequent when the parent accessions were more genetically distant^33^. Parents with rich genetic diversity are ordinarily selected for hybrid rice breeding to obtain the strongest heterosis^18^. By the end of June 2014, 2918 three-line hybrid rice cultivars had been released in China^34^. Such an amount of hybrid rice variability would produce numerous types of recombinants whose evolutionary direction would be unpredictable.

Establishment of hybrid rice progeny in the field depends mostly on volunteers. Farmers do not save the seeds of hybrid rice for future planting, as the benefits of the heterosis in the F_1_ hybrid are lost upon segregation in the F_2_ generation^18^. However, an estimated 300 kg ha^−1^ of rice seeds can reach the soil after harvesting due to natural shattering and losses during harvesting^35^. These seeds have the potential to establish as volunteers. Their success in subsequent rice plantings (in continuous rice production systems) or in rotational crops is closely related to climate conditions and crop production systems. Weedy rices with a filial relation to WA type hybrid rice were mostly detected in Hainan and Guangdong. The conditions of suitable temperatures and continuous rice cultivation in these southern provinces of China are conducive to weedy rice evolution and persistent generation of volunteers.

Indirect evolution of weedy rice would be mediated by gene flow between hybrid rice or its progeny and other rice varieties, wild relatives or already existing weedy types. The outcome of this process would be determined by the outcrossing frequency, the direction of the gene flow and the genetic background of the accompanying rice forms in the paddy field itself or in adjacent fields within the range of gene flow. A cultivar could serve as a bridge for the movement of the CMS gene from hybrid rice to weedy rice; recently it has been determined that some *indica* cultivars contain the CMS gene^31^. Outcrossing rates can significantly affect the heterozygosity of a population. The genes related to environmental adaptation and competitive ability present in different rice types would be selected for over time once initial hybridization provides opportunity for recombination^14^,^36^. Some genes for enhanced traits from rice, including transgenes, can introgress into weedy rice^36^,^37^,^38^,^39^,^40^. Likewise, undesirable weediness genes from weedy rice can be transferred into cultivated rice^41^. Independent studies have found that the F_1_, F_2_, and F_3_ progeny of hybrids between weedy rice and cultivated rice were at least as fit as the weedy rice parents, indicating that the hybrids have the potential to evolve into new types of weedy rice^16^,^42^,^43^,^44^. Thus, if weedy rice is present within or nearby hybrid rice fields, new weedy rice types may arise more rapidly through pollen exchange with existing hybrid rice.

Gene flow between cultivated rice and weedy rice can occur in both directions, with an outcrossing rate usually below 1%^10^,^35^,^45^,^46^. However, outcrossing may be affected by cultivar and weedy rice biotype^16^,^47^. Hybrid rice may have a higher outcrossing rate due to its exserted stigma^18^. Indeed the herbicide-resistant hybrid cultivar CLXL8 was a higher outcrosser than the conventional cultivar CL161^16^. Furthermore, hybrid rice for which a gametophyte abortion type sterile line is used, as in the case of CMS-BT, produces only approximately 50% viable pollen grains^18^, which could affect its outcrossing rate. The outcrossing rate is also determined by factors such as plant distance, plant height, wind direction, synchronization of flowering and pollen viability^35^,^46^,^48^,^49^. Hybrid rice volunteers segregate plants of diverse heights with extended flowering periods^18^,^50^. In addition, for hybrid rice that uses a sporophyte abortion type sterile line, as in CMS-WA, there will be male sterile individuals among the progeny that would have to be cross-pollinated by fertile individuals to create offspring.

There is also a risk of hybrid rice seed contamination with weedy rice seed, because the majority of hybrid rice seed is produced in open farm fields and not at experimental farms. A significant proportion of plants in a seed production field are a sterile line that must be cross-pollinated. Thus, without proper isolation or in the presence of weedy rice arising from dormant seed, there is opportunity for gene flow to occur with the consequence of off-types (weedy rice) being planted along when the obtained hybrid rice seed is sown^21^,^35^,^50^. This would accelerate the onset of the indirect evolution process previously discussed.

### Inferences on the time of origin of weedy rice

The proportion of weedy rice accessions found to have a direct relationship to hybrid rice by this study is much lower than expected, considering the high potential of hybrid rice to evolve into weedy rice. This propensity is likely because hybrid rice has heterozygous segregating progeny (volunteers) with a potentially higher outcrossing rate. Additionally, comparative studies of weedy and cultivated rice in China have shown that either weedy rice has a close relationship with companion cultivated rice or that the crop was involved in weedy rice evolution through gene introgression^10^,^11^,^12^,^13^,^17^. Thus, most of the current weedy rice accessions in China are related to conventional cultivated rice (including both landraces and conventional varieties). This poses the question of whether the current weedy rice biotypes evolved along with the introduction of hybrid rice or much earlier. Considering that hybrid rice and conventional cultivated rice have shared almost the same proportion of the rice cultivation area in China for several decades, one could surmise that most weedy rice accessions evolved from conventional cultivated rice much earlier, i.e., over thousands of years of rice cultivation. The worsening of the infestations currently observed is most probably related to agronomic factors, such as increased direct seeding, planting of contaminated seed and ineffective control measures. The regulations for rice seed certification in China address quality aspects including purity and germination, but weedy rice seed is not currently prohibited.

Furthermore, rice production in China has long depended on abundant landraces selected and kept by farmers since crop domestication began. In recent decades, landraces have been gradually replaced by improved varieties. During the Seventh Five-Year Plan period (1986-1990) of China, 34663 rice landraces were collected and stored, of which 8963 - 26% of the collection - had a red pericarp. Several red pericarp landraces were tall, matured early and were tolerant of poor soils and drought^51^. Historical records from Jiangsu also describe local rice cultivars as having long awns, a dark husk, a red pericarp and other traits frequently associated with weedy rice^52^. It is thus possible that landraces were an important source of the Rc (red pericarp) gene and of other weedy traits involved in weedy rice evolution.

Weedy rice continuously evolves with the cultivated rice it infests. Since hybrid rice cultivation is very recent, weedy rice evolving from it should be at an early stage of evolution. It is expected that with increased planting of hybrid rice, opportunities for weedy rice to directly or indirectly evolve from hybrid rice and containing the CMS gene will also increase. Long-term dynamics of weedy rice evolution from hybrid rice would also be modulated by outcrossing rates and by the contribution of Rf genes that are not always homozygous. Special attention should be paid if in the future, transgenic hybrid rice, particularly carrying herbicide resistance traits, is released, as its progeny might also have high outcrossing rates, and the new traits will alter the dynamics of weedy rice evolution under the selective pressure of the herbicide.

## Methods

### Weedy and cultivated rice materials

Thirty-six weedy rice accessions initially collected (12 each) in Liaoning, Jiangsu and Guangdong provinces were selected from the weedy rice germplasm bank of the Weeds Research Laboratory at Nanjing Agricultural University (NJAU) and used in field experiments for CMS detection (Supplementary Table S1, Figure S3). An additional 322 weedy rice accessions from 91 populations from 12 provinces where hybrid rice was or is widely planted were included in the molecular tests (Supplementary Table S2, Figure S4). All weedy rice samples were collected from populations infesting commercial rice fields by selecting a single panicle per plant of at least 20 plants separated by a distance of at least 10 m in each location. We randomly selected 1-5 individuals from these samples, representing each population. Eight pairs of sterile and maintainer rice lines (provided by the Rice Institute of NJAU) were used as parents for artificial hybridization and as controls for CMS markers (Table 1).

### General experimental approach

**Figure 6:**
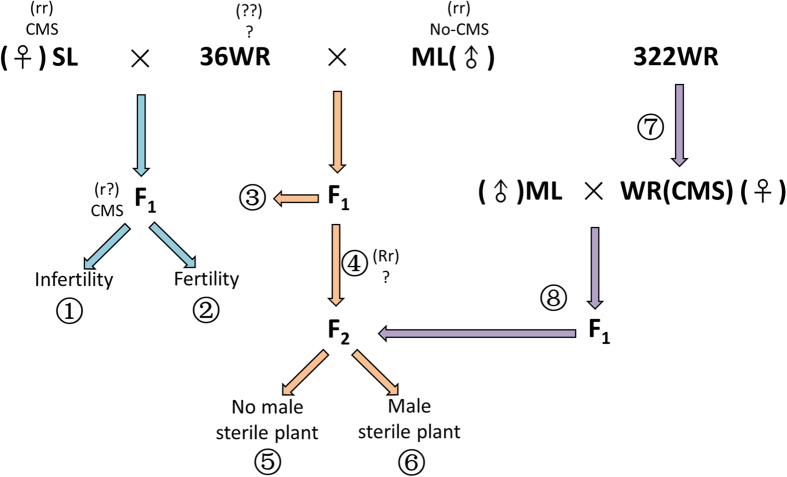
Schematic representation of the experimental crosses used to determine whether weedy rice accessions contained cytoplasmic male sterile (CMS) genes. SL: sterile line; ML: maintainer line; WR: weedy rice. ① The weedy rice parent contains neither restorer of fertility (Rf) genes nor CMS genes; ② The weedy rice parent contains Rf genes and might contain CMS genes; ③ F_1_ hybrid fertility (affinity) used to judge whether a false negative exists in the result of ① ④ Fertility segregation in F_2_ individuals used to detect which weedy rice parent contains Rf genes; ⑤ The weedy rice parent does not contain CMS genes; ⑥ The weedy rice parent contains a CMS gene; ⑦ Detection of a CMS gene in weedy rice by a CMS gene molecular marker; ⑧ Verification of the result by artificial hybridization.

Our experimental approach to determine whether hybrid rice is implicated as a direct source of some of the weedy rice biotypes currently infesting rice fields in China is illustrated in Fig. 6. It involved the detection in weedy rice of the most important CMS genes used in the production of hybrid rice cultivars as a diagnostic trait for their direct parenthood of weedy individuals. The *japonica* and *indica* affinity of the 36 weedy rice accessions was determined through morphological characterization and analysis of artificial crosses between the weedy forms and appropriate maintainer lines for pollen viability and seed setting. Artificial hybrids with sterile lines were also used to determine the presence of CMS and Rf genes in the weedy types.

### Artificial hybridization and fertility detection

The warm water emasculation method was used for artificial hybridization^53^. Selected panicles about to bloom were submerged for five minutes in warm water at 43 °C and 45 °C for *indica* and *japonica* rice, respectively, to kill the stamens without injuring the pistils. One-third to a half of each glume was carefully cut to expose the pistils. A paper pollination bag with both ends open was placed over each prepared panicle and closed at the bottom with a clip. While maintaining the opening at the upper end, pollen of the designated donor was sprinkled on the pistils, and the bag was closed.

The fertility of experimental materials was assessed by a pollen viability test and by determining the rate of seed set. Mature florets, with filaments about half the length of the glumes, were selected to detect pollen viability by I_2_-KI staining, either immediately or after being temporarily preserved in formaldehyde acetic acid solution. The stained samples were mounted on glass slides and observed by optical microscopy (Olympus Co., Tokyo, Japan). Three florets from each individual plant were selected. Each floret was observed in five vision fields containing approximately 100 pollen grains each. The ratio of stained to unstained pollen grains was determined. A healthy panicle was selected from each plant and self-crossed by enclosure in a porous non-woven fabric bag before flowering for determining seed set at maturity.

### Validation of a molecular marker of the male sterile gene

Fresh leaves were taken from each individual plant for total DNA extraction using plant genomic DNA rapid extraction kit (HF213-01, Yuanpinghao Biotech Co., Ltd. Tianjin, China). CMS-BT marker p3p4 (p3: 5′- ATGGCAAATCTGGTCCGATG-3′ and p4: 5′-ACTTACTTAGGAAAGACTAC-3′) and CMS-WA marker *cms* (*cms*F: 5′-ACTTTTTGTTTTTGTGTAGG-3′ and *cms*R: 5′-TGCCATATGTCGCTTAGACTTTAC-3′) were used. Polymerase chain reaction (PCR) was performed in an automated PCR apparatus (BioRAD PTC300) using a 25 μL amplification reaction system as follows: 1.0 μL DNA template at 10 ng μL^−1^, 12.5 μL of Premix Taq containing 0.625 U of DNA polymerase, 0.4 mM of each dNTPs and 2 × Taq buffer (TaKaRa Biotechnology Co., Dalian, China), 1.0 μL each of the forward and reverse primers at 10 μM and 9.5 μL of distilled water. The PCR procedure included an initial denaturation for 5 min at 94 °C; 30 cycles of 94 °C for 1 min, 55 °C for 1 min and 72 °C for 1 min; extension for 10 min at 72 °C and maintenance at 4 °C.

### Subspecies classification and affinity determination

Classification of weedy rice accessions into *indica* and *japonica* subspecies is important to better understand their genetic relationships with the accompanying cultivated rice. Subspecies identification was accomplished using the Cheng’s index^30^ protocol (Supplementary Table S3).

Sterile hybrids in crosses between sterile lines and weedy rice can occur because of *indica*-*japonica* cross-incompatibility or lack of the Rf genes in weedy rice. Thus, it was important to classify the accessions into the two subspecies and to test the affinity with typical *indica* (LTPB) and *japonica* (S28B) rices before detecting the Rf genes by artificial hybridization. The 36 weedy rice accessions were selected as female parents, and *indica* type WA maintainer line LTPB and *japonica* BT type maintainer line S28B were selected as pollen donors (72 hybrid combinations). Ten plants of each combination were used to determine pollen viability and seed setting rate. The differences in fertility of F_1_ hybrids (36WR × typical *indica*, 36WR × *japonica* rice) were used to determine the incompatibility between weedy rice accessions and sterile lines. These were used in the detection of the Rf genes to exclude false negatives and also to categorize the subspecies of weedy rice as *indica*- or *japonica*-like. Seed produced by selfing the F_1_ hybrids was used to detect CMS.

### Detection of cytoplasmic male sterility and restorer of fertility genes

Artificial hybridization is a reliable way to detect the presence of Rf and CMS genes in weedy rice accessions. Plants of the 36 weedy rice accessions were selected as male parents; WA type sterile line LTPA and BT sterile line S28A were used as female parents (resulting in 72 hybrid combinations). Pollen viability and seed setting rate of these F_1_ hybrids were determined. Weedy rice individuals without the Rf genes (those whose offspring pollen aborted and did not set seed) were eliminated because it was highly unlikely for them to carry the CMS-BT or CMS-WA.

The CMS-BT is gametophytic, and the F_2_ hybrid generation of a BT type male sterile line and a restorer line will only produce fertile offspring. Gene *orf79* is responsible for CMS-BT^54^. Marker p3p4 can amplify a 239 bp fragment which is part of *orf79* from the BT sterile line and its hybrid progeny, but cannot amplify a fragment in a maintainer line because it lacks CMS-BT^55^. Four pairs of BT sterile and maintainer lines were used to verify the reliability of this marker before using it to detect the CMS-BT in weedy rice.

The fertility of F_2_ progeny of which weedy rice was the female parent (containing the Rf genes for CMS-WA) and LTPB was male parent was determined. At least two hundred plants from each hybridized combination were randomly selected for tests of pollen viability and seed setting rate. Those plants identified as sterile were considered to be derived from the progeny of weedy rice accessions used as female parents containing the CMS-WA.

The molecular marker *cms* can amplify a 386 bp fragment from the WA sterile line and the derived hybrid, but not from the maintainer line^56^. It was selected for detecting the CMS-WA in the additional 322 weedy rice samples. The reliability of the *cms* marker was determined with WA sterile lines, WA maintainer lines and the weedy rice accessions confirmed to contain the CMS-WA. Artificial hybridization was also used to further guarantee the accuracy of detection with the molecular marker. Weedy rice accessions bearing the *cms* molecular marker for CMS-WA were used as female parents in crosses with LTPB as pollen parents. Fertility determination in the segregating F_2_ generation (at least two hundred plants from each hybridized combination) was used to assay whether the CMS-WA existed in the weedy rice parents.

### Corroboration of presence of cytoplasmic male sterility by specific molecular markers

In the course of analyzing the data and preparing the manuscript, *WA352* the gene responsible for CMS-WA was cloned^31^. In order to confirm the reliability of our procedures and the accuracy of our results, we used marker orWA352 (orWA352-F: 5′-GTTGATGGGTATGGATAGAG-3′ and orWA352-R: 5′-CGCAGGGCCTCGGTATATCTA-3′)^31^ based on *WA352* to detect the possible presence of CMS-WA in all 358 weedy rice samples.

## Additional Information

**How to cite this article**: Zhang, J. *et al*. Cytoplasmic-genetic male sterility gene provides direct evidence for some hybrid rice recently evolving into weedy rice. *Sci. Rep.* **5**, 10591; doi: 10.1038/srep10591 (2015).

## Supplementary Material

Supporting Information

## Figures and Tables

**Table 1:** Sterile and maintainer lines used as parents for artificial hybridization and verification of reliability of molecular markers.

**Sterile types**	**Sterile line**	**Maintainer line**
Wild abortive (WA)	LTPA	LTPB
	Tianfeng A	Tianfeng B
	Zhenshan 97A	Zhenshan 97B
	Zhenpin A	Zhenpin B
Boro type (BT)	S28A	S28B
	Liuqianxin A	Liuqianxin B
	863A	863B
	Xu II A	Xu II B
