# Supplementary material for: Cytoplasmic-genetic male sterility gene provides direct evidence for some hybrid rice recently evolving into weedy rice
Source: Sci Rep. 2015 May 27;5:10591. doi: 10.1038/srep10591 (PMC4445040; doi:10.1038/srep10591)
Supplement: Supporting Information [file srep10591-s1.pdf]

Supplementary information

Cytoplasmic-genetic male sterility gene provides direct evidence for some hybrid rice recently evolving into weedy rice

Jingxu Zhang[1], Zuomei Lu[2], Weimin Dai[1], Xiaoling Song[1], Yufa Peng[3], Bernal E. Valverde[1,4], Sheng Qiang[1*]

[1]Weed Research Laboratory of Nanjing Agricultural University, No.1 Weigang, Xuanwu District, Nanjing 210095, China; [2]State Key Laboratory of Crop Genetics &Germplasm Enhancement, Nanjing Agricultural University, No.1 Weigang, Xuanwu District, Nanjing 210095, China; [3]Institute of Plant Protection, China Academy of Agricultural Sciences, No.2 Yuanmingyuan West Road, Beijing 100193, China;[4]Faculty of Life Sciences, The University of Copenhagen, Hojebakkegaard Allé 13, Taastrup, DK-2630, Denmark.

Correspondence and requests for materials should be addressed to Sheng Qiang
E-mail: wrl@njau.edu.cn

Supplementary Table S1 Source of 36 weedy rice accessions for initial scrutiny of possible origin from commercial hybrid rice.

Sampling code	Abbreviation	Province	Source location (Population site)
WRLN001-01	LN1	Liaoning	Shenyang
WRLN002-01	LN2	Liaoning	Shenyang
WRLN003-01	LN3	Liaoning	Shenyang
WRLN004-02	LN4	Liaoning	Dandong
WRLN005-01	LN5	Liaoning	Dandong
WRLN006-02	LN6	Liaoning	Panjin
WRLN007-20	LN7	Liaoning	Panjin
WRLN008-01	LN8	Liaoning	Panjin
WRLN009-01	LN9	Liaoning	Tieling
WRLN010-01	LN10	Liaoning	Tieling
WRLN010-02	LN11	Liaoning	Tieling
WRLN010-03	LN12	Liaoning	Tieling
WRJS022-03	JS1	Jiangsu	Yangzhou
WRJS022-04	JS2	Jiangsu	Yangzhou
WRJS022-05	JS3	Jiangsu	Yangzhou
WRJS022-06	JS4	Jiangsu	Yangzhou
WRJS024-13	JS5	Jiangsu	Yangzhou
WRJS024-14	JS6	Jiangsu	Yangzhou
WRJS013-20	JS7	Jiangsu	Taizhou
WRJS014-16	JS8	Jiangsu	Taizhou
WRJS038-14	JS9	Jiangsu	Nantong
WRJS038-15	JS10	Jiangsu	Nantong
WRJS038-20	JS11	Jiangsu	Nantong
WRJS039-02	JS12	Jiangsu	Nantong
WRGD001-01	GD1	Guangdong	Zhanjiang
WRGD001-02	GD2	Guangdong	Zhanjiang
WRGD001-13	GD3	Guangdong	Zhanjiang
WRGD001-14	GD4	Guangdong	Zhanjiang
WRGD003-09	GD5	Guangdong	Zhanjiang
WRGD003-13	GD6	Guangdong	Zhanjiang
WRGD011-02	GD7	Guangdong	Zhanjiang
WRGD011-03	GD8	Guangdong	Zhanjiang
WRGD012-06	GD9	Guangdong	Zhanjiang
WRGD012-07	GD10	Guangdong	Zhanjiang
WRGD013-03	GD11	Guangdong	Zhanjiang
WRGD013-04	GD12	Guangdong	Zhanjiang

Supplementary Table S2 Source of additional 322 weedy rice accessions for scrutiny of possible origin from commercial hybrid rice

Population code (number)	Sampling number	Province	Source location (Population site)
WRJS007	4	Jiangsu	Lianyungang
WRJS013	4	Jiangsu	Taizhou
WRJS014	4	Jiangsu	Taizhou
WRJS015	4	Jiangsu	Taizhou
WRJS023	4	Jiangsu	Yangzhou
WRJS024	4	Jiangsu	Yangzhou
WRJS025	4	Jiangsu	Yangzhou
WRJS026	4	Jiangsu	Yangzhou
WRJS027	4	Jiangsu	Yangzhou
WRJS035	4	Jiangsu	Taizhou
WRJS036	4	Jiangsu	Yangzhou
WRJS037	4	Jiangsu	Nantong
WRJS038	4	Jiangsu	Nantong
WRJS044	4	Jiangsu	Changzhou
WRJS045	4	Jiangsu	Changzhou
WRJS046	4	Jiangsu	Changzhou
WRJS047	4	Jiangsu	Yangzhou
WRJS049	4	Jiangsu	Taizhou
WRJS050	4	Jiangsu	Nantong
WRJS051	4	Jiangsu	Changzhou
WRJS052	4	Jiangsu	Nantong
WRJS053	4	Jiangsu	Yangzhou
WRJS054	4	Jiangsu	Yangzhou
WRJS055	4	Jiangsu	Yancheng
WRJS056	4	Jiangsu	Suqian
WRJS057	4	Jiangsu	Yancheng
WRJS058	4	Jiangsu	Yancheng
WRJS059	4	Jiangsu	Yangzhou
WRJS060	4	Jiangsu	Taizhou
WRJS061	4	Jiangsu	Zhenjiang
WRJS062	4	Jiangsu	Yangzhou
WRJS063	4	Jiangsu	Lianyungang
WRJS064	4	Jiangsu	Lianyungang
WRJS065	4	Jiangsu	Xuzhou
WRJS068	4	Jiangsu	Yancheng
WRJS069	4	Jiangsu	Yancheng
WRJS070	4	Jiangsu	Huaian
WRJS072	4	Jiangsu	Taizhou
WRJS074	4	Jiangsu	Nanjing
WRJS075	4	Jiangsu	Nanjing

WRJS076	4	Jiangsu	Changzhou
WRJS077	4	Jiangsu	Suzhou
WRJS078	4	Jiangsu	Wuxi
WRJS079	4	Jiangsu	Nanjing
WRSH002	3	Shanghai	Chongming
WRAH005	3	Anhui	Wuhu
WRAH017	3	Anhui	Maanshan
WRHN001	3	Henan	Xinyang
WRJX005	3	Jiangxi	Shangrao
WRZJ007	3	Zhejiang	Shaoxing
WRHU007	3	Hunan	Changde
WRHU011	3	Hunan	Yiyang
WRYN001	3	Yunnan	Kunming
WRYN007	3	Yunnan	Dali/Dali
WRGZ003	3	Guizhou	Anshun
WRGX001	3	Guangxi	Liuzhou
WRGD001	3	Guangdong	Zhanjiang
WRGD002	3	Guangdong	Zhanjiang
WRGD003	3	Guangdong	Zhanjiang
WRGD004	3	Guangdong	Zhaoqing
WRGD005	3	Guangdong	Zhaoqing
WRGD008	3	Guangdong	Maoming
WRGD009	3	Guangdong	Maoming
WRGD011	3	Guangdong	Zhanjiang
WRGD012	3	Guangdong	Zhanjiang
WRGD013	4	Guangdong	Zhanjiang
WRGD016	3	Guangdong	Zhanjiang
WRGD022	5	Guangdong	Zhanjiang
WRGD023	5	Guangdong	Zhanjiang
WRGD024	3	Guangdong	Yangjiang
WRGD025	3	Guangdong	Zhaoqing
WRGD026	3	Guangdong	Zhanjiang
WRGD027	3	Guangdong	Zhaoqing
WRGD028	3	Guangdong	Zhanjiang
WRGD029	3	Guangdong	Yangjiang
WRGD030	3	Guangdong	Zhanjiang
WRGD031	3	Guangdong	Zhanjiang
WRGD032	3	Guangdong	Zhanjiang
WRGD033	3	Guangdong	Zhaoqing
WRGD034	3	Guangdong	Zhaoqing
WRHA008	3	Hainan	Dongfang
WRHA009	3	Hainan	Dongfang
WRHA010	3	Hainan	Dongfang
WRHA011	3	Hainan	Dongfang
WRHA013	3	Hainan	Lingshui
WRHA014	3	Hainan	Lingshui

WRHA015	3	Hainan	Lingshui
WRHA016	3	Hainan	Yanzhou
WRHA018	3	Hainan	Qionghai
WRHA019	3	Hainan	Wenchang
WRHA020	3	Hainan	Haikou

Supplementary Table S3 The index characters and their scoring standards of the Cheng's Index Method

Character	Score/character state				
	0	1	2	3	4
Glume hairiness	Short, regular	Short, less regular	Nearly glabrous	Long, irregular	Long, overlapped
Phenol reaction	Dark	Light	Very light	Trace	None
Interval between 1st and 2nd node of panicle axis, cm	1.5	2.0	2.5	3	3.5
Glume color at heading	Greenish white	Whitish green	Yellowish green	Light green	Green
Leaf pubescence	Very dense	Dense	Medium	Few	No
Length-width ratio of spikelets	4.0	3.5	3.0	2.5	2.0

Subspecies type is judged by totaling the scores of the six indexes, 0~8 considered as *indica* type, 9-13 for *indica*-cline type , 14-17 for *japonica*-cline type, 18-24 for *japonica* type.

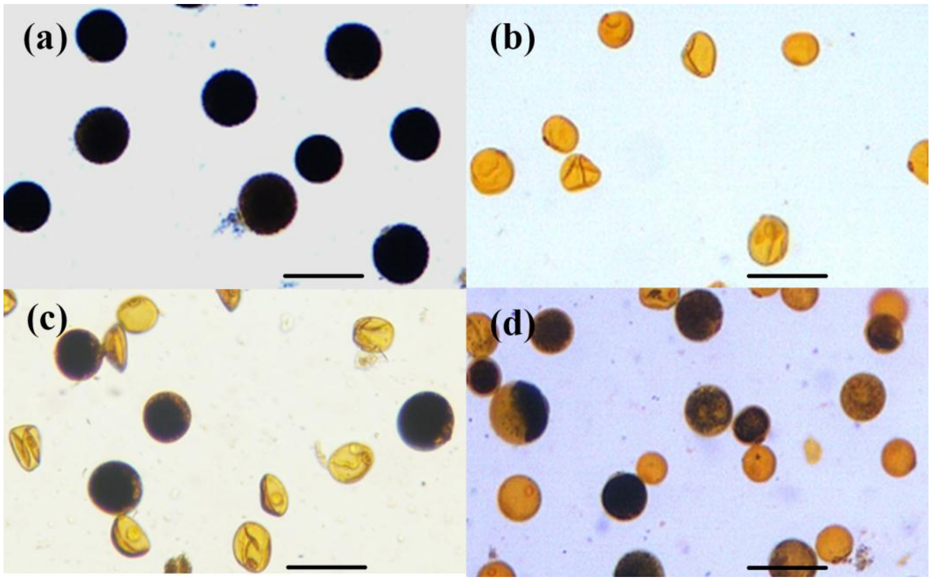

Supplementary Figure S1 Morphology of pollen grains used to determine their viability. (a) Normal fertile pollen grains stain black and exhibit similar particle size; (b) Aborted pollen grains stain yellow and are deformed; (c) Easily distinguished fertile and aborted pollen grains in hybrid progeny for which LTPA was the female parent and weedy rice was the male parent; (d) Difficult-to-distinguish fertile and aborted pollen grains in hybrid progeny for which S28A was the female parent and weedy rice was the male parent, because the presence of the Boro-type (BT) abortive pollen grains with spherical shape, different particle size and staining. Pollen grains were stained with 1% I_2-KI solution. Bars, 50 µm.

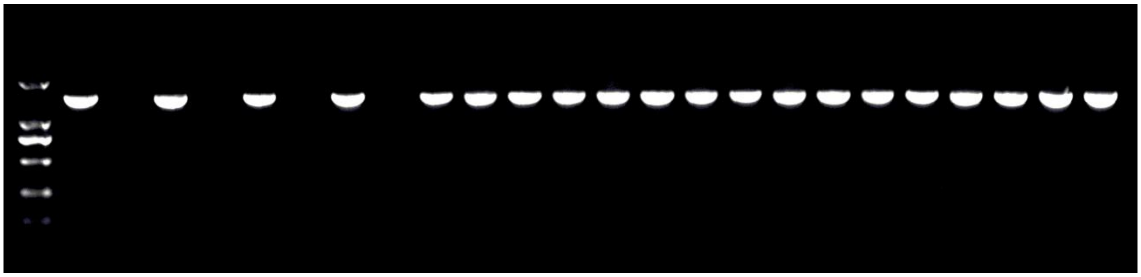

Supplementary Figure S2 Corroboration of results obtained by *cms* marker using orWA352. Marker orWA352 only amplifies a 1432bp fragment from samples containing CMS-WA. Four wild abortive (WA) type sterile line samples and sixteen weedy rice accessions were confirmed to contain CMS-WA. The results corresponded to those obtained by the *cms* marker. From left to right: Marker (D2000 DNA ladder), Zhenshan97A, Zhenshan97B, Tianfeng A, Tianfeng B, Zhenpin A, Zhenpin B, LTPA, LTPB, WRJS026-01 (Jiangsu), WRHA016-01, WRHA016-03 (Hainan), GD9, GD10, GD11, GD12, WRGD012-04, WRGD013-06, WRGD013-13, WRGD013-17, WRGD022-03, WRGD022-06, WRGD022-09, WRGD022-10, WRGD022-13 (Guangdong). Sequenced amplified products contained the *WA352* gene.

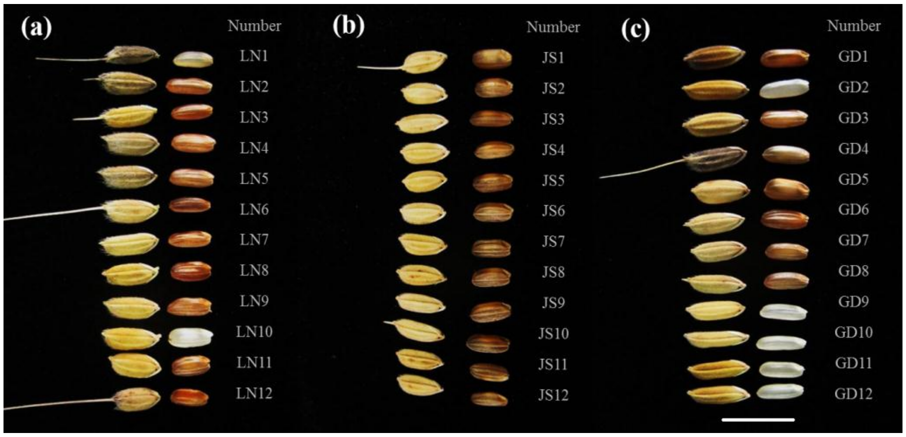

Supplementary Figure S3 Caryopses of 36 weedy rice populations from three provinces in China. (a) Liaoning; (b) Jiangsu; (c) Guangdong. Bars, 10mm.

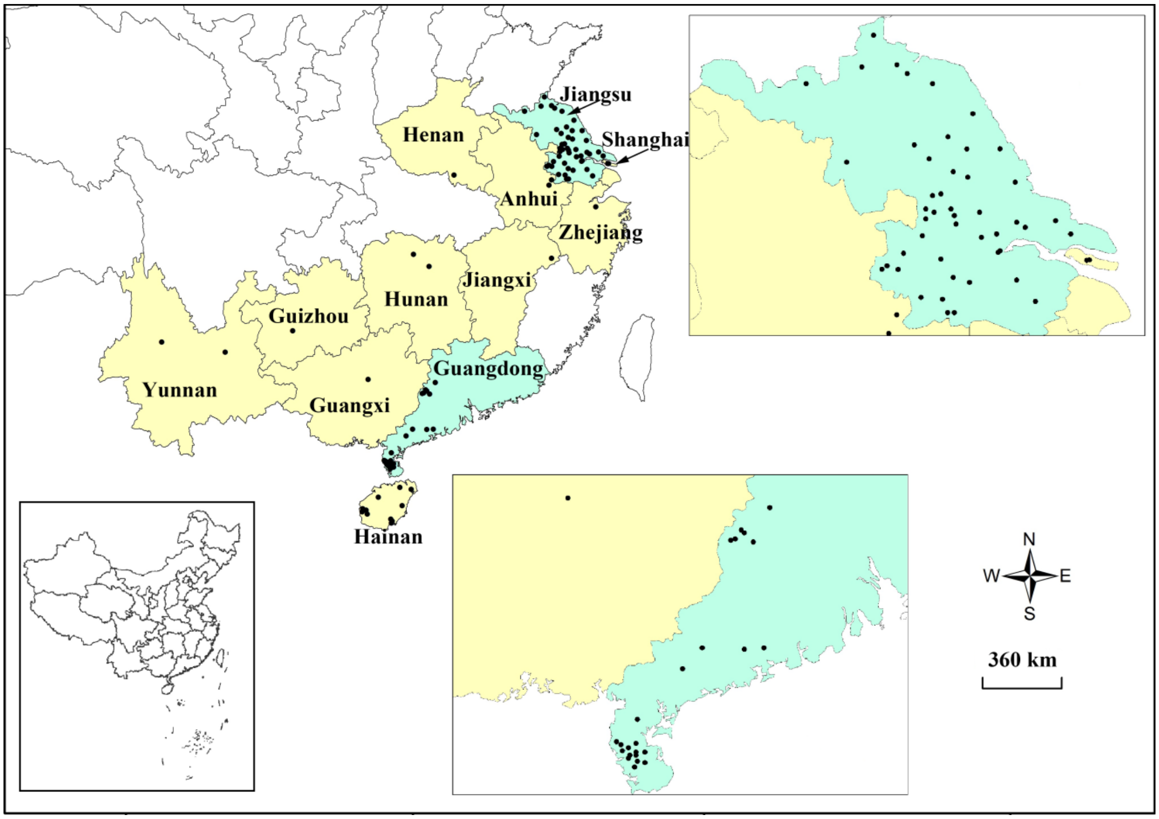

Supplementary Figure S4 Collection sites of 91 weedy rice populations in 12 provinces where hybrid rice is widely planted. Maps generated using ArcGIS 10.0.
